# Fluid-Solid Interaction Simulation Methodology for Coriolis Flowmeter Operation Analysis

**DOI:** 10.3390/s21238105

**Published:** 2021-12-03

**Authors:** Evgeniia Shavrina, Vinh-Tan Nguyen, Zeng Yan, Boo Cheong Khoo

**Affiliations:** 1 Mechanical Engineering, National University of Singapore, Singapore 117565, Singapore; mpekbc@nus.edu.sg; 2 Institute of High Performance Computing, Singapore 138632, Singapore; nguyenvt@ihpc.a-star.edu.sg; 3 National Metrology Centre, Singapore 138634, Singapore; zeng_yan@nmc.a-star.edu.sg

**Keywords:** Coriolis flowmeter, turbulence model, fluid-structure interaction, validation

## Abstract

Numerical simulation is a widely used tool for Coriolis flowmeter (CFM) operation analysis. However, there is a lack of experimentally validated methodologies for the CFM simulation. Moreover, there is no consensus on suitable turbulence models and configuration simplifications. The present study intends to address these questions in a framework of a fluid-solid interaction simulation methodology by coupling the finite volume method and finite element method for fluid and solid domains, respectively. The Reynolds stresses (RSM) and eddy viscosity-based turbulence models are explored and compared for CFM simulations. The effects of different configuration simplifications are investigated. It is demonstrated that the RSM model is favorable for the CFM operation simulations. It is also shown that the configuration simplifications should not include the braces neglect or the equivalent flowmeter tube length assumption. The simulation results are validated by earlier experimental data, showing a less than 5% discrepancy. The proposed methodology will increase the confidence in CFM operation simulations and consequently provide the foundation for further studies of flowmeter usage in various fields.

## 1. Introduction

Coriolis flowmeters (CFMs) are widely used in various industries for the accurate measurement of mass flow rate. The accuracy of the CFM metering is aimed to be higher than 99.9% for a water flow [1,2]. However, Cheesewright et al. [3] showed that, for example, a pulsating flow may lead to an erroneous meter reading. Moreover, a disturbed flow before a CFM may cause a flowmeter output reading deviation of up to 4% [4]. Furthermore, Weinstein [5] stated that the multiphase presence may cause a significant error in industrial applications of CFMs. Hence, the effects of flow features on CFMs should be further investigated to ensure the desired accuracy.

Numerical simulation is considered a promising tool for the study of the flow features impact on a CFM operation. However, this simulation is not trivial due to the working principle of this flowmeter. A typical Coriolis flowmeter contains three major components as generally described in the literature [6]. The first is one (or more) tube (s) through which the measured fluid passes. The second major component is a transmitter that oscillates the tube (s) at a natural frequency. The fluid accelerates inside the flowmeter tube as it flows toward the maximum amplitude point, and slows down as it passes that point. The tube twist, which is caused by this change of the flow speed, is measured as a time shift by the electromagnetic sensors, which are the third major component of a CFM. Additionally, the braces on the flowmeter tube (s) may be present, increasing the complexity of the flowmeter configuration. Hence, the working principle of CFMs is based on the mutual influence between the fluid flow and flowmeter tube. Consequently, this introduces challenges in the CFM operation modelling.

One of the earliest methodologies for the CFM simulation was presented by Sultan and Hemp [7], where the flowmeter was described by Euler vibrating beams interacting with 1D fluid flow [8]. The described methodology was successfully validated by experimental data for oil and water flows [7]. However, the Euler beam theory is inapplicable for complex geometries due to the discretization of the simulation domain by straight elements. Hence, the application range of this method is limited to the simple CFM configurations as highlighted by Sultan and Hemp [7]. Moreover, the effects of flow features on the flowmeter output cannot be investigated by this methodology.

Binulal et al. [9] presented an improved methodology for the CFM modelling, which can be applied for the CFMs of complex configurations, such as omega and U-shaped CFMs. This methodology employed the Timoshenko beam theory and finite three-node elements for the fluid description [8]. Although this approach overcame some of the limitations of the methodology by Sultan and Hemp [7], it is only suitable for a steady flow without pulsations and other flow features deemed important.

Belhadj et al. [10] presented a methodology for the CFM simulation, which employed ANSYS FE software for the description of the fluid influence on the flowmeter tubes [10]. While the demonstrated method was suitable for the rapid assessment of the flow pulsation influence on the CFM operation, it was not possible to take into account other flow features, such as multiphase presence and flow velocity profile change, due to the simplification of the fluid behavior.

Bobovnik et al. [11] presented a more complicated simulation methodology to investigate the effects of fluid features on the CFM operation. The combination of the finite volume and finite element methods, also known as a fluid–solid interaction (FSI) analysis, was introduced in this study for the CFM simulation [11]. The flow was described by Reynolds-averaged Navier-Stokes (RANS) equations and the CFM tube was modelled as a shell structure. At the same time, the implicit coupling approach was employed to connect the tube and fluid modellings [11].

The model proposed by Bobovnik et al. [11] was applied to study the effects of the fluid flow on the CFM operation by other researchers. For example, Enz [12] investigated the time shift dependence on the velocity profile by the simulation based on this model. In addition, Hu et al. [13] investigated the influence of the fluid viscosity on the CFM operation by the FSI model based on the methodology by Bobovnik et al. [11]. Kumar and Anklin [14] studied the Reynolds number influence on the CFM operation by the FSI simulation. Finally, the modification of this FSI methodology was used by Athanase [15] and Romanov and Beskachko [16] to study the CFM operation during the multiphase metering and the influence of fluid dissipation characteristics on the CFM modelling, respectively. However, different turbulence models were applied in these studies to complement the RANS equations [11,13,15,16,17]. The shear stress transport (SST) turbulence model was employed by Athanase [15], Hu et al. [13], and Romanov and Beskachko [16], while Bobovnik et al. [11,18], Kumar and Anklin [14], Hua et al. [19] and Enz [12] used the k-ε turbulence model. In addition, Bobovnik et al. [20] conducted a comparison of the Reynolds stress model (RSM) and k-ε models and reported an insignificant difference. However, it should be noted that Bobovnik et al. [20] used a straight-tube CFM, which is expected to experience the influence of the turbulence models less. Hence, so far, there is no consensus on the turbulence model selection for the simulation of the U-shaped or omega-shaped tube CFM operation.

It should be highlighted that a few studies included the comparison of the modelling results with experimental data. Among the methodologies which can describe the influence of fluid flow features on CFM operation, only qualitative comparisons were provided by Belhadj et al. and Kumar and Anklin [10,14]. The quantitative validation was conducted by Hu et al. [13], but the relative error of the calculated time shift equaled 25%. Moreover, Kolhe and Edlabadkar [21] demonstrated a quantitative validation of the CFM simulation for the laminar flow, again reporting a relative error of up to 25%. Therefore, there is a lack of satisfactorily validated simulation methodologies which limits confidence in the numerical analysis of the CFM operation.

Moreover, the acceptable simplifications of the flowmeter configuration were not determined so far for the simulation of the CFM operation. In general, only the flowmeter tube is modelled, while the masses of sensors and the driver, as well as the tube braces, are neglected [7,9,10,11,16,17]. However, these configuration features may influence the simulation accuracy, as was suggested by Sultan and Hemp [7]. Furthermore, Bobovnik et al. [18,22] suggested that the consideration of braces as well as the masses of sensors and the driver is necessary. At the same time, Kumar and Anklin [14] and Taluja et al. [23] included braces in the simulation but not the masses of sensors and the driver. Hence, there is no consensus if it is necessary to consider the braces and the masses of sensors and the driver in the simulation.

Overall, there is a need for the simulation methodology to investigate the dynamics of the fluid flow and its effects on the CFM operation. While several such methodologies have been presented, there is no consensus on the choice of the turbulence model and the acceptable configuration simplifications for the CFM simulation. The present study compares the eddy viscosity turbulence (SST and SST with curvature correction (SST-CC)) and RSM turbulence models, as it has not been done for U-shaped CFMs before. This comparison is not only necessary for the CFM investigations but contributes to a general discussion on the application range of the turbulence models. Moreover, the present study determines if the configuration simplifications, namely the neglect of braces and the masses of sensors and driver, as well as the concept of equivalent length, are acceptable. In addition, there are a lack of comprehensive validations of numerical simulation methodologies for the CFM operation with the experimental data.

The current work aims at bridging the above mentioned gaps in numerical analysis of CFMs. In this study, it has been shown that the most accurate simulation of the CFM operation is obtained by the RSM model in comparison with the SST and SST-CC models. This comparison provides further input to the ongoing discussion on the application range of turbulence models and their limitations. It has also been shown that the braces and the masses of sensors and driver should be taken into account for the CFM simulation. At the same time, the equivalent length concept should not be employed for a high accuracy simulation. Finally, the presented methodology is validated with a generally acceptable error against two sets of independent experimental data, providing confidence in the methodology. Hence, the proposed methodology will allow for a reliable and comprehensive numerical investigation of the CFM operation. Consequently, this will facilitate the investigation of the various flow features’ influence on the CFM accuracy, for example multiphase flow characteristic such as in cryogenic liquid, in future studies.

## 2. Methods

This section describes the methodology and the assumptions accepted for the set of numerical simulations of the Coriolis flowmeter. This set includes the simulations with the different configuration simplifications: the equivalent tube length and the neglect of the braces and the masses of the driver and sensors; and different turbulence models: the SST, SST-CC and RSM models. The simulations are conducted in the ANSYS software package, including ANSYS CFX and ANSYS Mechanical. The following aspects of the CFM simulation are considered and described: flowmeter configuration, FSI coupling, governing equations, boundary conditions, and mesh discretization. The assumptions for each of these aspects of the simulation are provided.

### 2.1. Flow Meter Configuration

**Figure 1:**
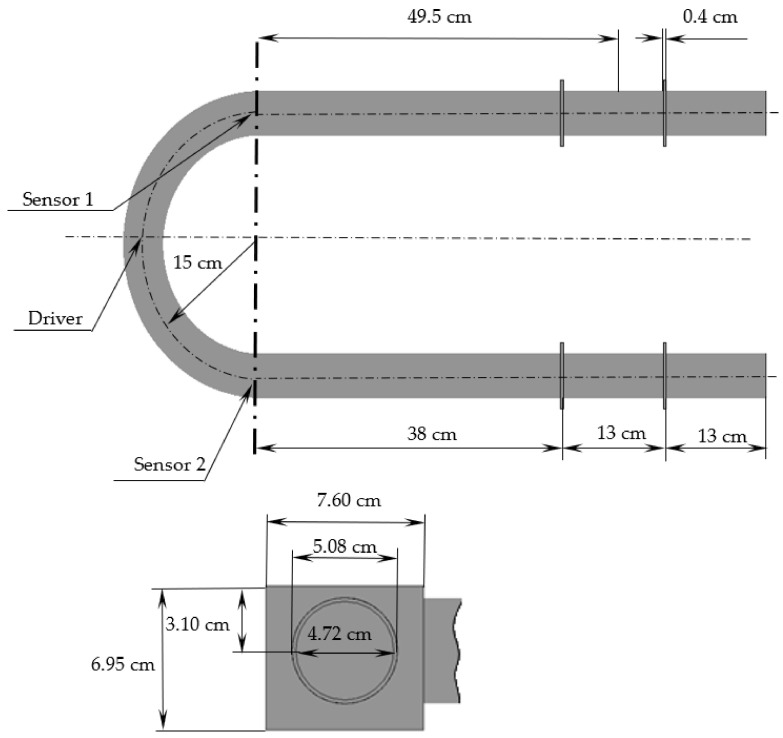
The Coriolis flowmeter dimensions [7,24].

The CFM configuration, which was used in the present study, was experimentally studied by Sultan, and Sultan and Hemp [7,24]. The investigated flowmeter consisted of two U-shaped tubes, four braces, two sensors and one driver. In the present study, only one tube was considered to decrease the computational time, as it can be assumed that the motions of the tubes do not influence each other. The dimensions of the modelled CFM are presented in Figure 1. It should be noted that due to the error in the dimensions of the braces, which were provided by Sultan and Sultan and Hemp [7,24], the braces’ dimensions were measured from other images in the same study [24]. In the setup used, the tube material was stainless steel 316 and the working fluid was water [7]. It should be noted that standard stainless steel parameters were used, which may be different in reality due to the manufacturing variation. The stainless steel tube was assumed to be smooth. The properties of the tube and fluid are listed in Table 1.

As was concluded by Wang et al. [25], the difference between the CFM operations at free and forced vibrations is negligible. Moreover, it is a common practice to use a forced vibration for the CFM simulation if the signal processing nuances are not investigated in the study [12,15,20,26]. Finally, the frequency and the amplitude are fixed in the experimental study by Sultan and Hemp [7] which was used for the validation of the simulation. Hence, the flowmeter tube oscillated at the driver’s location following Equation (1) [24].

(1) $$A=A_{0}\cdot sin\left(2\pi \cdot f\cdot t\right)$$

where $A_{0}\;$ is the amplitude of the CFM tube oscillation which equals 25 μm, *f* is the frequency of CFM tube oscillation and equals 72.31 Hz, *A* is the position of CFM tube, and *t* is the time.

**Figure 2:**
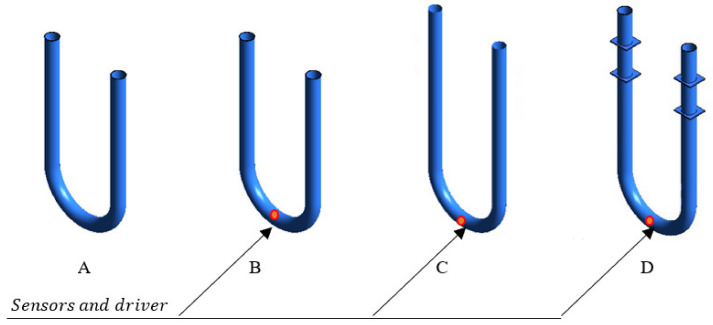
(**A**): equivalent length, the mass of sensors and driver is not considered, with braces not being taken into account; (**B**): equivalent length, the mass of sensors and driver is considered, with braces not being taken into account; (**C**): full length, the mass of sensors and driver is considered, with braces not being taken into account; (**D**): full length, the mass of sensors and driver is considered, with braces being taken into account.

As shown in Figure 2, four cases of different CFM configuration simplifications were investigated in the present study to determine their acceptability. Firstly, the equivalent tube length, which equals 49.5 cm (Figure 1), may be used instead of a real CFM tube length according to Sultan and Hemp [7]. The equivalent length is the tube length which provides a natural frequency, equal to the natural frequency of the CFM tube with braces and the mass of sensors and driver [7]. The application of this concept decreases the computational cost through model simplification. The equivalent length application is represented by the Case A in this study (Figure 2). The mass of the sensors and the driver, which equals 0.4 kg [21], is taken into account for Cases B, C, and D (Figure 2). In addition, the full length of the flowmeter tube is considered for Case C. Finally, we not only use the full length of the CFM tube, but the braces are also taken into account for Case D.

### 2.2. Numerical Analysis Methodology

Fluid-solid interaction (FSI) simulation is utilized to analyze the CFM operation in the present study. Since the fluid is restricted by the solid domain, the moving boundary method [27] is applied instead of the immersed boundary method [28], which is used for the solid surrounded by liquid. Generally, the explicit and implicit FSI approaches are differentiated [29]. While the explicit approach finds the future state from the present [30], the implicit method solves both states in a single matrix [31]. This difference requires a much smaller time step for the explicit FSI, which leads to the increase in the calculation time. Therefore, the implicit approach is preferable and used in this study. The implicit approach may be classified into monolithic and iterative implicit FSI approaches in general. Due to the fact that the monolithic solution is time- and resource-consuming in comparison with the iterative implicit method [32], the iterative implicit approach was used in this study to decrease the computational cost without loss of accuracy.

The dynamic three-dimensional spring-mass system equation [33] has been used to describe the tube structural behavior during the CFM operation. The right side of the equation is determined by the fluid influence on a solid domain. The gravitational force is taken into account, and it is assumed that the tube’s ends are fixed. Moreover, a symmetry boundary condition is applied to simulate half of each brace. The obtained Equation (2) is solved by using ANSYS mechanical software [33]:

(2) $$\left[M\right]\left\{\ddot{u}\right\}+\left[K\right]\left\{u\right\}+\left[F\right]=\left[F^{fluid}\right]$$

where $\left[M\right]$ is the mass matrix, $\left[K\right]$ is the stiffness matrix, $\left\{\ddot{u}\right\}$ is the acceleration vector, $\left\{u\right\}$ is the deformation vector, [*F*] is the applied load vector, and $\left[F^{fluid}\right]\;$ is the fluid load vector.

The Newtonian fluid flow through the modelled CFM is governed by Navier-Stokes equations [34]. The fluid is considered incompressible as this is a common simplification for the simulation of water metering by CFM [12,13,21,27,35,36]. Since the Reynolds number based on the CFM diameter exceeds 4000 for the investigated velocity range, the flow in the CFM tube is in the turbulent regime. Hence, turbulence modelling was applied to mitigate the computational cost of the direct solution of the Navier-Stokes equations. The statistical turbulence model or Reynolds-averaged Navier-Stokes (RANS) approach was employed to represent the averaged flow quantities and model all the scales of turbulence effects [37]. Hence, RANS Equations (3) and (4) are solved for the fluid domain.

(3) $$\frac{\partial U_{j}}{\partial x_{j}}=0$$

(4) $$\rho \frac{\partial U_{i}}{\partial t}+\rho \frac{\partial }{\partial x_{j}}\left(U_{j}U_{i}\right)=-\frac{\partial p}{\partial x_{i}}+\frac{\partial }{\partial x_{j}}\left(\tau_{ij}-\rho \bar{u_{i}u_{j}}\right),$$

where *t* is the time, *U* is the velocity, *x* is the geometrical coordinate, *p* is the pressure, *τ* is the molecular stress tensor, and *ρ* is the fluid density.

The averaging of the flow quantities in the RANS approach introduces additional terms, namely the Reynolds stresses [37]. These terms may be calculated by the application of the eddy viscosity or Reynolds stress (RSM) models. The eddy viscosity models are usually applied for CFM simulations [13,15,16,17]. These models employ Boussinesq’s hypothesis, which assumes that the Reynolds stresses are related to the mean velocity gradients and eddy viscosity [38]. Hence, the Reynolds stresses are calculated following Equation (5):

(5) $$-\rho \bar{u_{i}u_{j}}=\mu_{t}\left(\frac{\partial U_{i}}{\partial x_{j}}+\frac{\partial U_{j}}{\partial x_{i}}\right)-\frac{2}{3}\left(\rho k+\mu_{t}\frac{\partial U_{k}}{\partial x_{k}}\right)\delta_{ij}$$

where *U* is the velocity, *x* is the geometrical coordinate, $\mu_{t}$ is the turbulence viscosity, *δ* is the identity matrix of the Kronecker delta function, *k* is the kinetic energy, and *ρ* is the fluid density.

Among eddy viscosity models, the SST model is commonly used for the CFM simulation [13,15,16] because it combines the advantages of the k-ε and k-ω models and includes the accurate treatment of the near-wall region [39]. However, the eddy viscosity turbulence models, including the SST model, are generally insensitive to the Coriolis force and streamline curvature effects, leading to the typical simulation error of 20% [40,41,42,43]. Due to the fact that the velocity fields in a U-shaped CFM tube are determined by the tube curvature [18], alternative descriptions of the Reynolds stresses provided by the Reynolds stress model (RSM) and the SST model with a curvature (rotational) correction (SST-CC) are also utilized in this study.

Compared to the eddy viscosity model, which employs algebraic equations to describe the Reynolds stresses, the RSM model solves the differential transport equations for six Reynolds stress components. This difference shows the theoretical advantage of the RSM model application in rotating systems and for the flow curvatures. The rotation is directly described in the last term of Equation (6) [44].

(6) $$\frac{\partial \rho \bar{u_{i}u_{j}}}{\partial t}+\frac{\partial }{\partial x_{k}}\left(U_{k}\rho \bar{u_{i}u_{j}}\right)\;=\frac{\partial }{\partial x_{k}}\left(\left(\mu +\frac{\mu_{t}}{\sigma_{k}}\right)\frac{\partial \bar{u_{i}u_{j}}}{\partial x_{k}}\right)+P_{ij,b}-\frac{2}{3}\beta^{\prime }\rho k\omega \delta_{ij}+P_{ij}+\Phi_{ij}+\rho \cdot \Omega_{k}^{rot}\cdot (\tau_{jm}\varepsilon_{ikm}+\tau_{im}\varepsilon_{jkm})$$

where *t* is the time, *U* is the velocity, *x* is the geometrical coordinate, $\beta^{\prime }$ is the *k-ω* turbulence model constant, *ω* is the angular velocity, $\Omega_{k}^{rot}$ is the rate of frame rotation, $\varepsilon_{m}\;$ is the Levi-Chivita factor, Φ is the pressure-strain correlation, $\sigma_{k}\;$ is the turbulence model constant for k equation, *ρ* is the fluid density, *μ* is the dynamic viscosity, $P_{b}\;$ is the buoyancy turbulence production term, and *P* is the shear turbulence production term.

In general, there are two different variants of the RSM model: *ε*-based and baseline (BSL) RSM models. The BSL RSM model, which has been discussed in detail by Gerolymos and Vallet [45], was applied in the present study due to a more accurate treatment of the near-wall region. In addition, the SST model with a curvature (rotational) correction (SST-CC) [46] was investigated as a middle step between the RSM and SST models. This model is based on the SST model but includes a simplified and generalized RSM model rotational component [46].

The high-order resolution advection scheme was applied to ensure sufficient accuracy and reduce the computational cost. In particular, a blend factor, which is a key part of the advection term calculation, varies throughout the domain depending on the local solution field [47]. The transient term in the fluid domain is discretized by the implicit second-order backward Euler scheme. The first and the last time step values were approximated by this scheme [48]. This scheme is robust, implicit, conservative in time, and does not have a time step limitation [48].

### 2.3. Computational Mesh and Boundary Conditions

**Figure 3:**
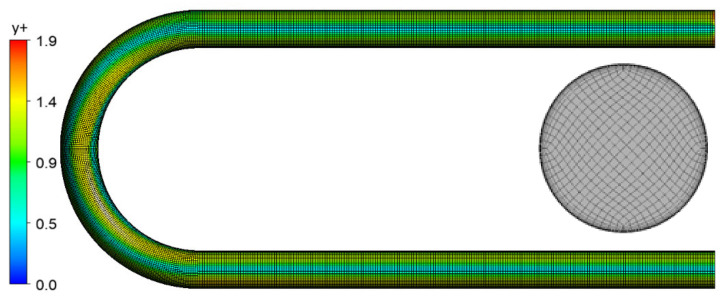
A typical space discretization of the fluid domain and y+ distribution for 8 m/s flow.

In the present study, the governing equations for the fluid flow were solved by the finite volume method using ANSYS CFX software. The computational domain was discretized using the hexahedral mesh with 13 near-wall layers for the description of the boundary layer in this study, as shown in Figure 3. The mesh orthogonal quality was above 0.95 and the mesh aspect ratio was less than 20. It should be noted that the y+ value was less than two to enable the low-Reynolds near-wall formulation in all conducted simulations [47], as depicted in Figure 3.

**Figure 4:**
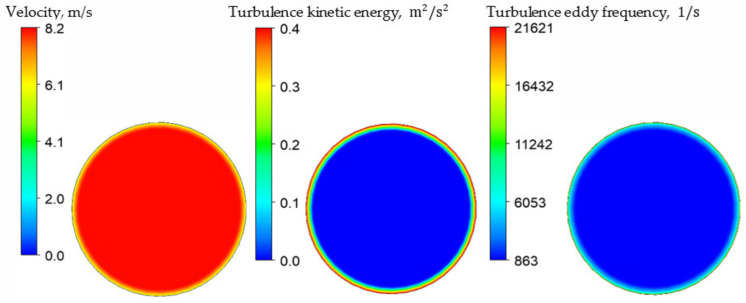
A typical profile set used as an inlet condition.

The no-slip boundary condition was applied on the walls of the flowmeter tube, and the low-Reynolds near-wall formulation was used. The velocity, turbulence kinetic energy, and turbulence eddy frequency profiles of fully developed flow in the pipe were used as an inlet boundary condition, following the description of the experimental setup by Sultan [24]. The profiles shown in Figure 4 were obtained by the simulation of a straight pipe with the same cross-sectional area and prescribed inlet mass flow rate while the turbulence intensity was set equal to 5%. It should be highlighted that the change of the profile generally does not influence the operation or the error of a U-shaped CFM, as indicated by Sultan and Bobovnik et al. [18,24]. At the same time, static atmospheric pressure was applied as an outlet boundary condition, following the description of the experimental setup by Sultan [24]. The constant temperature of 20 °C was used for the investigation as this temperature was used in the experimental study by Sultan [24]. However, it should be noted that Sultan mentioned the possible temperature variation of ±5 °C, which should be taken into account as a potential error source. Finally, a steady-state simulation was carried out to provide an initial condition for the subsequent transient analysis in all present simulations.

## 3. Results

### 3.1. Numerical Verification

**Figure 5:**
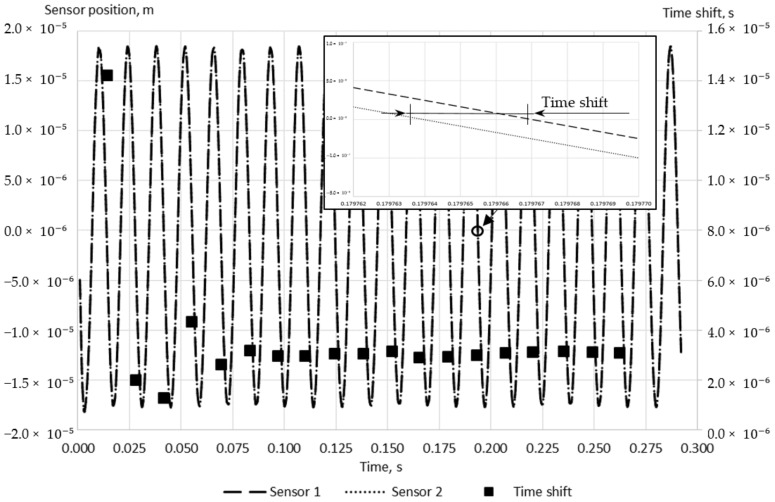
A typical change of the calculated tube position at sensors location and time shift depending on time (1 m/s, Case D).

A typical change of the calculated tube position at sensors location with time is demonstrated in Figure 5. The time shift is measured at the intersection of the calculated wave function and the zero line, as in the experimental study conducted by Sultan [24]. Since the time shift is a key parameter in this study, it is monitored in addition to the conservation of mass and momentum and maximum residuals to ensure the simulation convergence. The imbalances of the conservation are kept in the range of 0.5% and the maximum residuals are less than 1 × 10^−5^ for the fluid domain for all conducted simulations. The time shift convergence criteria, which is less than 3% of the relative change with time for five sequential time shifts, is fulfilled for all discussed simulations as well. A typical simulation time, which is required to meet the selected convergence criteria, equals 0.25 s, which is equivalent to approximately 19 oscillation periods.

The investigation of the space discretization influence on the simulation results was conducted to verify the proposed numerical approach. Case A was selected as an example and the SST turbulence model was applied as the first approximation since this is a conventional model for the CFM simulations [13,15,16]. The time shift, which is the key characteristic of the CFM tube twist, was calculated and analyzed by the application of the grid convergence index, which is a standardized way to describe the mesh convergence quality [49]. As demonstrated in Table 2, the time shift is converged when the number of mesh elements exceeds 168,000. Therefore, the mesh of 168,000 elements was used to conduct the simulations in the present study, with the discretization error of 2%.

In addition, the study of the time step influence on the simulation results was conducted. Again, the Case A in combination with the SST turbulence model was used for this investigation. It has been shown earlier that a sufficient time step approximately equaled one-twentieth of the period [10,14] for CFM simulations. In the present work, the time steps of 5 × 10^−4^, 1 × 10^−3^ and 2 × 10^−3^ s were considered. As demonstrated in Table 3, the time shift and the CFM sensitivity, which is a time shift divided by the mass flow rate [50], are found to converge at the time step equaled to 1× 10^−3^ s, which indicates around 14 steps per oscillation period. In a way consistent with earlier work, this time step was applied in the present study to provide sufficient accuracy at an acceptable computational cost.

### 3.2. The Investigation of the Linearity between Flow Speed and Time Shift

**Figure 6:**
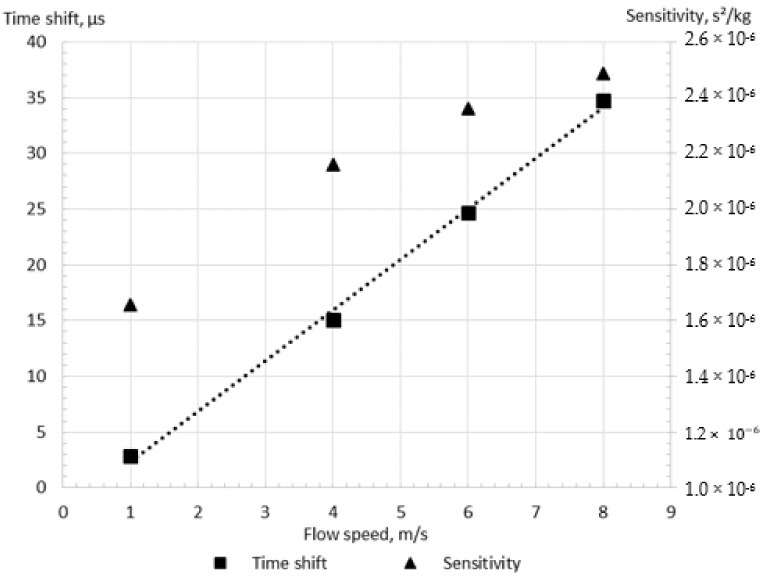
The linearity of the relationship between flow speed and time shift for the Case A, the shear stress turbulence model.

The relationship between the mass flow rate and the time shift is linear for CFMs, as indicated by Wang and Baker [51] and Sharma et al. [26]. The simulations were conducted using the Case A with the SST turbulence model for different flow speeds to verify this feature of the flowmeter. As demonstrated in Figure 6, the operation of the CFM was analyzed at flow speeds equal to 1 m/s, 4 m/s, 6 m/s, and 8 m/s. It may be noted that indeed the time shift grows with the mass flow rate as expected for the CFM. The R-squared coefficient of the obtained trend line equals 0.999, confirming the linearity of the present numerical results, with the fitting error of 0.1%. Because of the confirmed linear relationship between the flow speed and the calculated time shift, two flow speeds are assumed to be sufficient for the CFM operation investigation subsequently. It should be noted that a significant zero shift, equal to 0.5 m/s, is observed, causing the change of the flowmeter sensitivity with the flow speed, as shown in Figure 6. The variation of the sensitivity, which is the time shift divided by the mass flow rate, with the flow rate has also been observed in previous studies by Hu et al. [13] and Enz [12]. The causes of this phenomenon are investigated in the present paper.

### 3.3. The Investigation of Acceptable CFM Configuration Simplification

**Figure 7:**
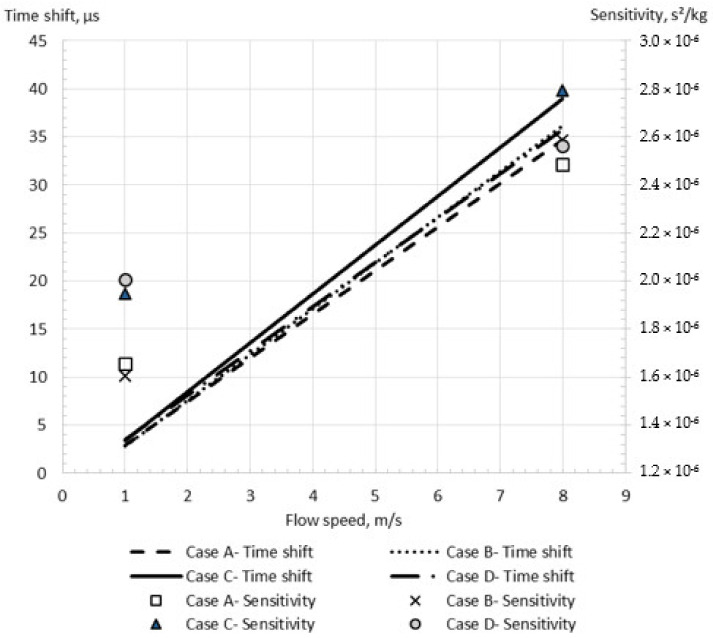
Simulation results for different geometrical simplifications for the shear stress turbulence model.

The simplification of the device configuration is an important part of any modelling methodology due to the significant influence on the simulation accuracy and computational cost. In the present study, four cases of configuration simplifications, as shown in Figure 2, were investigated. By comparing Cases A and B, it was found that the consideration of sensors and driver mass in the simulation changes the time shift on 3.2–4.0% at the flow velocity of 1–8 m/s, as shown in Figure 7. At the same time, the equivalent length assumption and the braces’ neglect lead to a more significant change in the numerical results. Indeed, while the difference between the time shifts obtained by the simulations with equivalent length assumption (Case B) and full configuration description (Case D) is small for high flow rate (1% at 8 m/s flow velocity), this error becomes 20% at 1 m/s flow velocity. At the same time, the neglect of braces (Case C versus Case D) may increase the calculated time shift by up to 8% at 8 m/s flow speed, while it changes only by 2.8% at 1 m/s speed. Indeed, the braces increase the stiffness of the flowmeter tube, leading to a smaller time shift for Case D in comparison with Case C.

**Figure 8:**
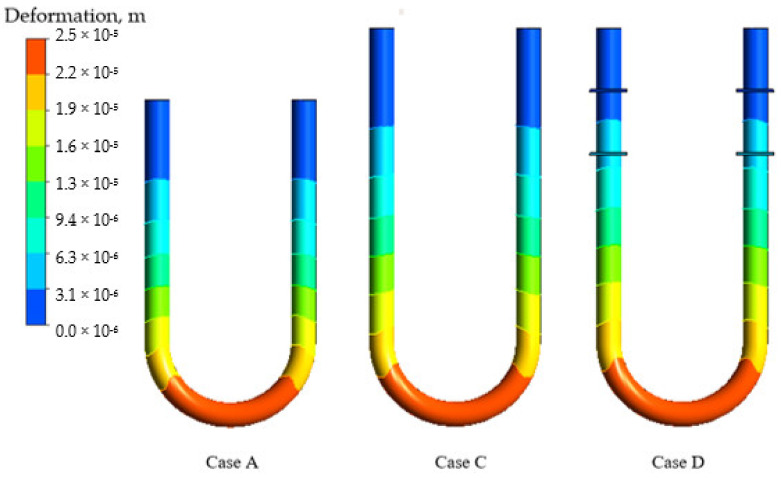
Tube deformation for 8 m/s for the shear stress turbulence model.

The impact of the equivalent tube length assumption and the braces neglect on the CFM operation simulation is investigated by the analysis of the tube deformation fields shown in Figure 8. According to Sultan and Hemp [7], the equivalent length, which is a CFM tube segment experiencing deformation with or without braces, equals 49.5 cm. However, this is a significant underestimation of the deforming tube part length, as demonstrated in Figure 8. In addition, the deformation of the tube is significantly influenced by braces, which increase the stiffness of the system, as shown in Figure 8 for the Case D. Hence, the previously proposed configuration simplification [24], which uses equivalent length and neglects braces, is not advisable for a reliable FSI modelling of CFM operation. Instead, the description of the flowmeter geometry should be as detailed as possible and any neglect of the flowmeter components, such as braces, should be avoided. Therefore, the Case D, which considers the full tube length, braces, and mass of sensors and drivers, is the only acceptable configuration and is further used in the present study.

### 3.4. The Investigation of Turbulence Models Effects

Two turbulence models—RSM and SST—are investigated for CFM analysis application, and the simulation results are validated by the experimental data provided by Sultan [7]. Sultan [7] investigated the commercial CFM, which was described in Section 2.1 of the present paper. The direct measurement of the time shift was conducted for the CFM in the water rig at various flow rates by Sultan [7], which is controlled by a reference meter.

**Figure 9:**
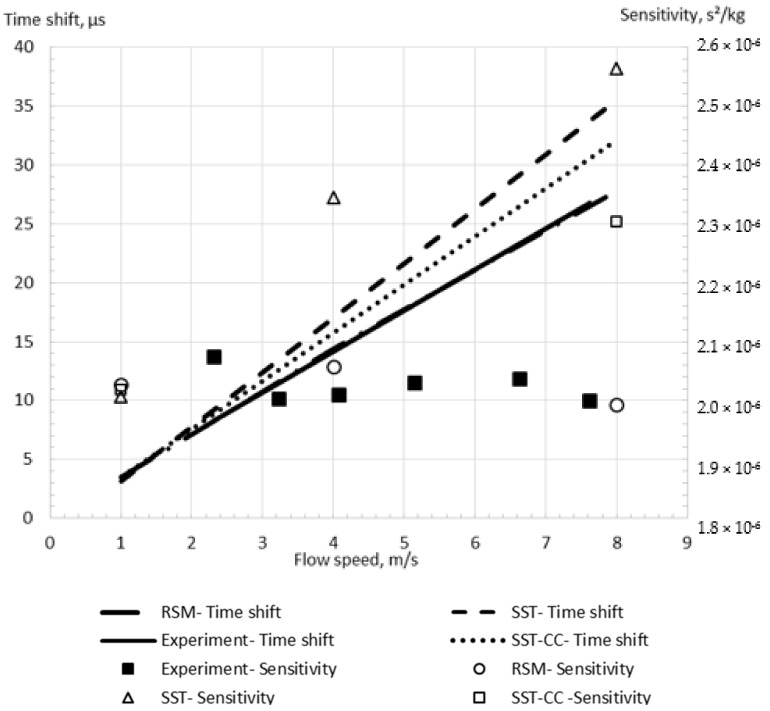
The simulation results for the shear stress (SST) and Reynolds stress (RSM) turbulence models for the Case D in comparison with experimental data.

The difference between the RSM and SST models predictions is insignificant for the low flow speed equal to 1 m/s, as shown in Figure 9. Indeed, the relative errors of the calculated time shifts equal 3.2% and 4.3% in comparison with experimental data for the SST and RSM models, respectively at 1 m/s. However, the error from using the SST model is equal to 25.5% at the higher flow speed of 8 m/s, while the RSM model provides an acceptable error of 2.5% (Figure 9). Additionally, the SST model modification which includes the curvature correction [46] (SST-CC) is studied. While the relative error of the time shift obtained by the SST model with the curvature correction (SST-CC) equals 4% in comparison with the experimental data for 1 m/s flow, this error achieves 13% for a high flow speed. This value is between the values calculated by the SST and RSM models, confirming that the difference between the turbulence models results is caused by the description of the rotational flow component due to the wall curvature. Moreover, it shows that while the SST-CC model improves the description of the flow along the wall curvature, it is less accurate than the RSM model. This is explained by the fact that the SST-CC model simplifies the rotational flow component.

It can be seen that the RSM model more accurately predicts the sensitivity change with the flow speed, preventing the artificial zero shift observed for the SST model. Indeed, the change in the sensitivity with the flow speed predicted by the RSM model is less than 3%, while this change is up to 14% for the SST model, as demonstrated in Figure 9. It should be noted that the sensitivity slightly varies with the flow speed in the experimental study by Sultan and Hemp [7], as demonstrated in Figure 9. Because of this and the fact that the simulation flow speed values are not exactly the same as the experimental values, it is hard to calculate the exact sensitivity error. Through the comparison of the sensitivity values at the two closest flow speeds between the simulations (4 m/s and 8 m/s) and the experiments (4.1 m/s and 7.6 m/s), the errors could be approximated to be 16% and 21% for the SST model, and 2% and 0.5% for the RSM model.

**Figure 10:**
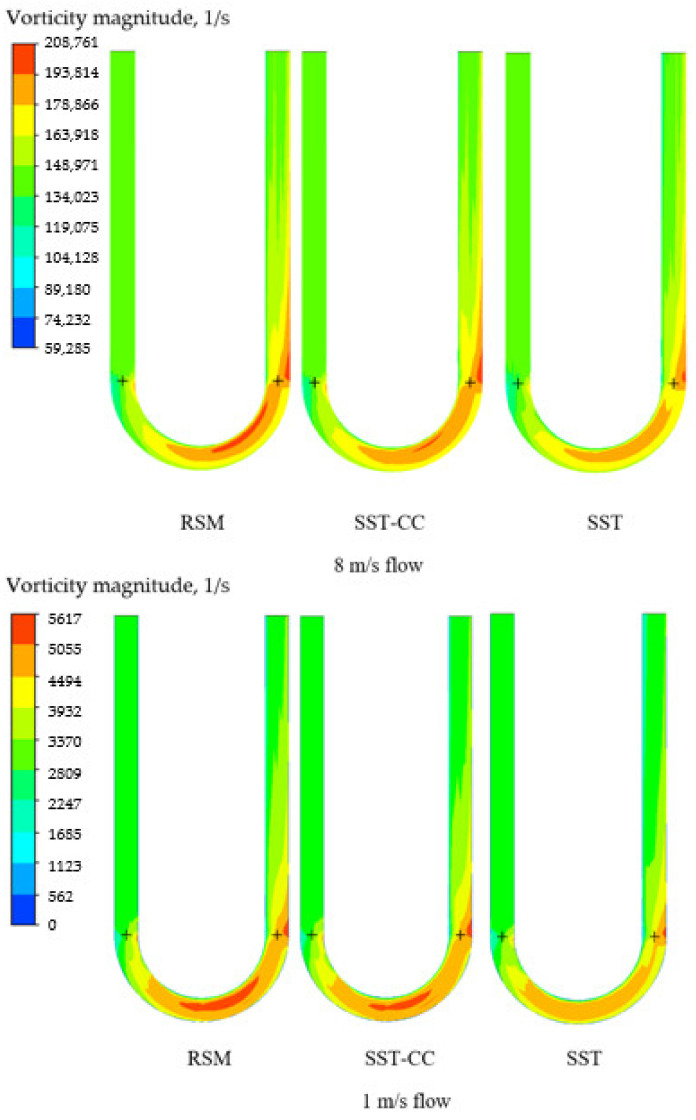
Vorticity magnitude values for the shear stress (SST) and Reynolds stress (RSM) turbulence models at different flow rates.

The difference between the SST and RSM models predictions is attributed to the ability of the RSM model to capture a lower turbulence intensity upstream in the flowmeter tube. This is demonstrated by the vorticity magnitude values in Figure 10. As may be seen the SST model predicts higher vorticity than the RSM model especially for a higher flow speed. This is due to the fact that the magnitude of the Coriolis force is proportional to the linear velocity. It may be noted that, again, the values calculated by the SST-CC model are between the values predicted by the SST and RSM models.

**Figure 11:**
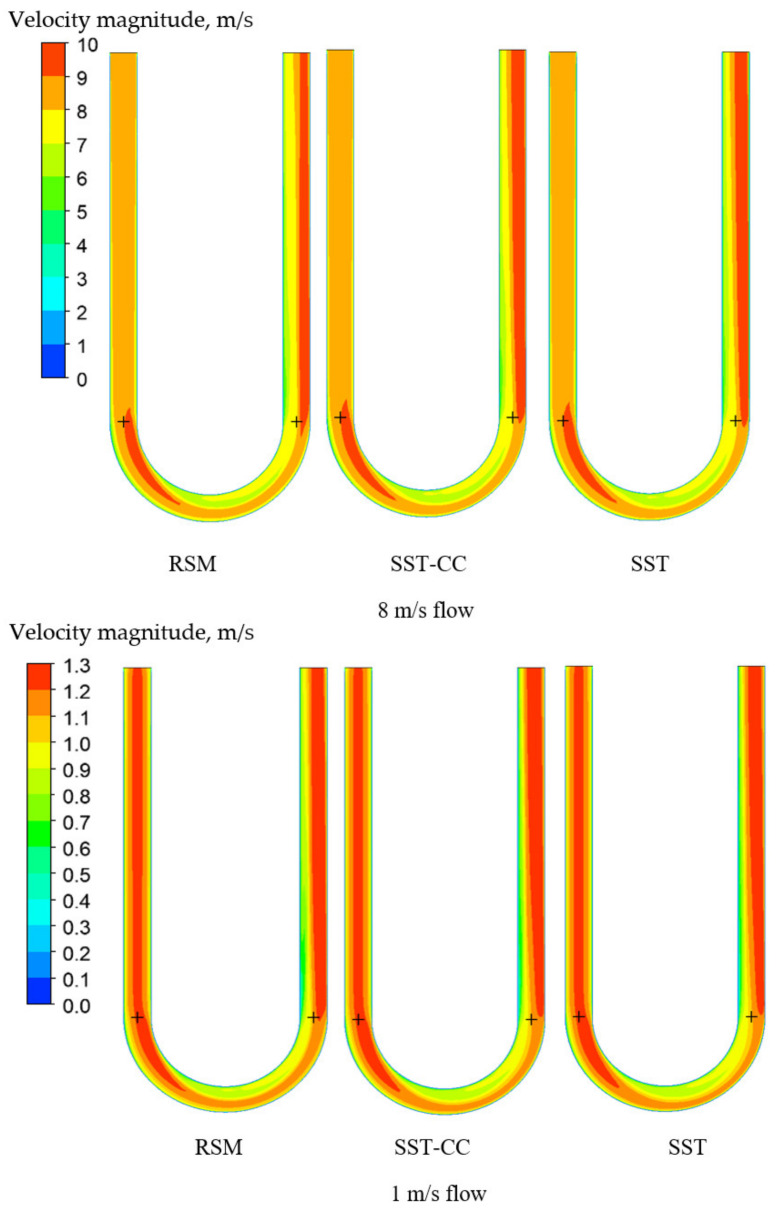
Velocity fields for the Case D for the shear stress (SST) and Reynolds stress (RSM) turbulence models at different flow rates.

Consequently, this leads to a larger flow separation region in the outlet part of the tube for the RSM model (Figure 11) which is in agreement with the observations by Luo and Lakshminarayana [52]. As demonstrated, the separation region predicted by the SST-CC model is smaller than predicted by the RSM model but larger than described by the SST model. This is due to the fact that while the SST-CC model takes into account the rotational component, it simplifies it. The correct estimation of the flow separation region is important for the overall CFM operation understanding. It is especially important for the CFM application in multiphase flow metering as larger separation region leads to a more non-uniform bubbles distribution, potentially causing a larger metering error.

**Figure 12:**
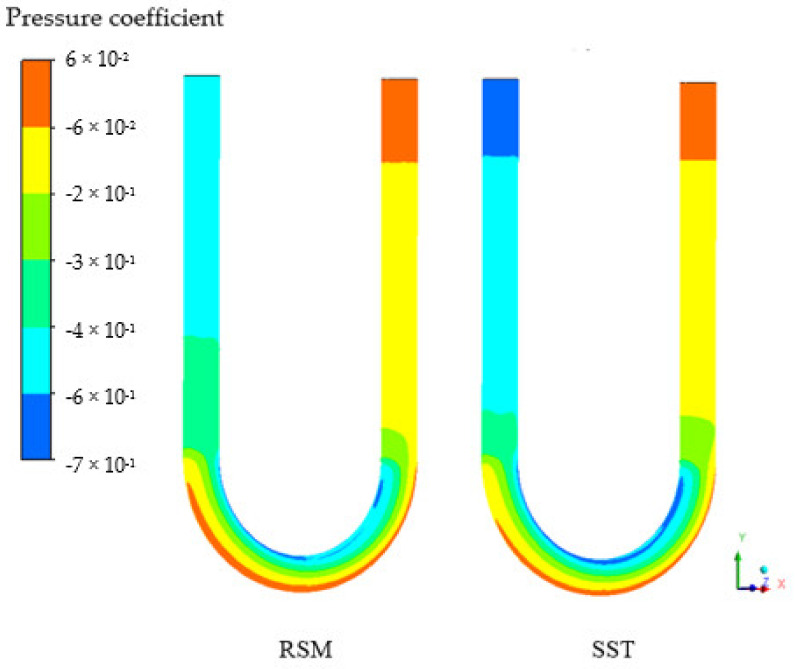
The pressure coefficient for the shear stress (SST) and Reynolds stress (RSM) turbulence models for the Case D at 8 m/s.

Due to the linkage between the flow separation and the effective through-flow area, the respective pressure coefficient distribution for these turbulence models ensues, as shown in Figure 12, attesting to the findings by Luo and Razinsky [42]. The pressure coefficient is determined following the study by Chang [53]. Since the pressure of the fluid on the wall is directly related to the deformation of the tube, the time shift, which characterizes the tube deformation, is lower for the RSM model.

**Figure 13:**
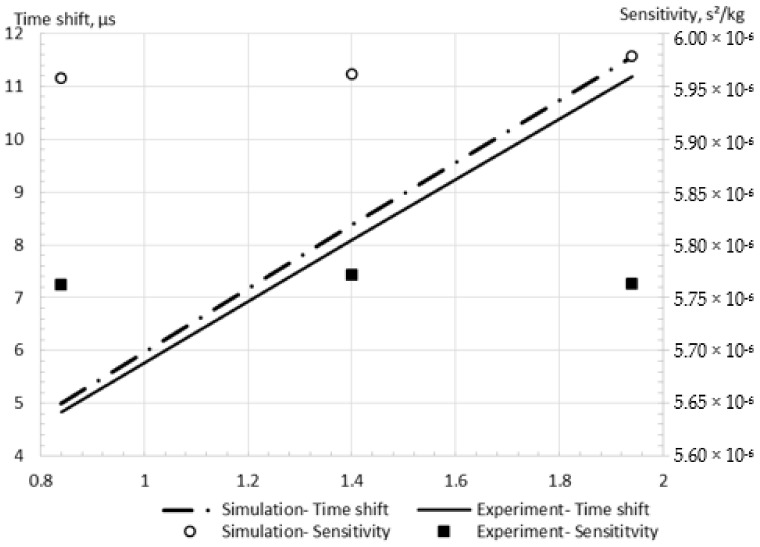
Simulation results in comparison with experimental data by Hu et al. [13].

In summary, the RSM model combined with the minimal neglect of configuration features should be applied for a reliable simulation of the CFM operation. An additional validation study is conducted to increase the confidence in the proposed approach. The dual U-shaped CFM, which is described and experimentally studied by Hu et al. [13], is used for this investigation. It should be highlighted that while this CFM was U-shaped, the same as the CFM which was used by Sultan [7], there was a difference in the exact dimensions of the CFMs, which were described by Hu et al. [13]. Hu et al. [13] investigated the CFM operation in the TAF certified flow testing factory, which includes a gravimetric setup, providing confidence in the measurements. Moreover, it should be noted that the temperature was strictly controlled and was set to 25 °C. The time shift was measured experimentally by RS-485 [13], providing the necessary data for the comparison with the experiment. The relative error of the calculated time shift is less than 4% for pure water flow rate from 0.84 kg/s to 1.94 kg/s in comparison with the experimental data (Figure 13). Furthermore, the sensitivity errors between the experiments [13] and the simulations (RSM model) are calculated for the validation. It is seen from Figure 13 that the errors are equal to 3.5%, 3.3%, and 3.8% at the mass flow rates of 0.84 kg/s, 1.4 kg/s, and 1.94 kg/s, respectively. It may be noted that the order of the obtained error equals the order of the simulation error for the previous validation case by Sultan [7]. The present error may be explained by manufacturing variation of the material properties and numerical errors, which are described in detail in Section 3.5. Therefore, it is reconfirmed that the proposed methodology is reliable for the CFM operation simulation.

### 3.5. The Error Analysis

To further understand the reliability of the simulation results, the quantified errors are analyzed and summarized in Table 4. First, the tube material properties may vary, depending on the manufacturer. According to the references [54,55], the errors of Young’s modulus, density, and shear modulus are calculated to ±3.80%, ±1.25%, and ±5.13%, respectively. This may partially explain the relative error of the calculated time shift in comparison with experimental results. It should be highlighted that the investigation of the influence of those variations on the simulation results requires significant computational time. Therefore, they are only listed here but the final impact on the simulation results is not quantified.

In addition, the numerical calculation introduces the errors to the results. As demonstrated in Table 4, the fitting error for the simulation results’ linearity is ±0.1% according to Section 3.2. Moreover, the discretization error is present and equal to ±2% as shown in Section 3.1.

Moreover, the experimental measurements conducted by Sultan are characterized by the errors from two main sources: time measurement of ±0.01% and reference meter of ±0.26% [24]. Furthermore, the possible temperature variation of ±5 °C leads to ±3% time shift error in the experimental study by Sultan [24]. It should be noted that this error is not present in the experimental study by Hu et al. [13] as the temperature was strictly controlled. Similarly, the temperature is also set as a constant value in the present simulation. Hence, the effect of the temperature variation is not considered in the present study, despite the fact that the temperature influences CFM characteristics, [56] which needs further investigation.

It is worth noting that several assumptions are made which do not influence the simulation accuracy according to the authors’ best knowledge. For example, only one of two flowmeter tubes is simulated as it is assumed that the motions of these tubes do not influence each other. Moreover, the flowmeter tube is considered smooth and the fluid is assumed to be incompressible. In addition, the dimensions of the braces are provided by Sultan [24] with a mistake, hence, as indicated above, they are measured from other images of the braces in the same study [7]. Moreover, the forced CFM oscillation is used in the conducted simulation as it is considered as an acceptable assumption in other studies [25].

## 4. Discussion

In the present study, the methodology for the fluid-solid interaction (FSI) simulation of the Coriolis flowmeter (CFM) operation was developed and validated by available experimental data. First, the influence of the flowmeter configuration simplifications on the results of the CFM operation simulation was investigated. It was shown that the equivalent flowmeter tube length assumption and the neglect of braces significantly influence simulation results. Hence, the braces should be taken into account and the full length of the tubes should be considered for any numerical investigation of a CFM operation.

Secondly, the effects of different turbulence models on the CFM simulation were discussed. It was shown that the baseline Reynolds stress model (BSL RSM) provides better accuracy compared with the eddy viscosity-based shear stress turbulence (SST) model for the CFM operation analysis. This is due to the fact that the BSL RSM model captures the flow curvatures more accurately in comparison to the eddy viscosity models.

Finally, the developed simulation methodology, which takes into account the braces’ presence and is based on the BSL RSM turbulence model, was validated by the experimental data provided by Hu et al., and Sultan and Hemp [7,13]. The difference between the calculated and experimentally obtained time shifts is less than 5%. The same order of accuracy was achieved for the calculation of CFM sensitivity since it is directly related to the time shift value. The existing error may be explained by the variance of stainless steel mechanical properties. Moreover, the errors from the experimental study, possible temperature variation in one set of experimental data, the discretization error and the results linearity fitting errors may be considered as minor contributors to the observed difference between the simulation and experimental results.

Overall, the proposed FSI simulation methodology will be a robust approach for subsequent CFM studies. The successful validation with experimental data provides confidence in this methodological application, which was not available before. Due to the fact that this methodology takes into account the flow features effects on the flowmeter operation, it may be used for the investigation of pulsating, bubbly, and other flows, which are common in industrial applications of CFM. Consequently, a better understanding of these flow effects will increase the reliability of the CFM application in various fields. It should be noted that while this methodology allows for the investigation of the temperature change influence, multiphase metering effects, flow compressibility, velocity profile impact, etc., it was not validated for the change of these parameters and requires further investigation.

## Figures and Tables

**Table 1:** Tube and fluid properties [21].

Flowmeter Part	Property	Value
Tube	Shear modulus (GN/m^2^)	80
	Density (kg/m^3^)	8027
	Young’s modulus (kg/m^2^)	208
Fluid	Density (kg/m^3^)	1000

**Table 2:** Space discretization study for 8 m/s flow.

Number of Elements	Time Shift, ∆*t*, μs	Relative Difference, %	Grid Convergence Index, %
168,000	37.7		
352,000	34.7	8	9
712,000	35.3	2	4

**Table 3:** Time discretization study for 8 m/s flow.

Time Step (s)	Time Shift, ∆*t* (μs)	Relative Difference of Time Shift (%)	$\mathrm{Sensitivity}\;(s^{2}/kg)$
2 × 10^−3^	38.8	11.80	2.78 × 10^−6^
1 × 10^−3^	34.7	2.96	2.49 × 10^−6^
5 × 10^−4^	33.7		2.42 × 10^−6^

**Table 4:** Errors for simulations and experiments.

	Sources		Error (%)
Simulations	Variation of tube material properties	Young’s modulus	±3.80
		Density	±1.25
		Shear modulus	±5.13
	Numerical error	Linearity fitting	±0.10
		Discretization	±2.00
	Time measurement		±0.01
Experiments	Reference meter		±0.26
